# Pulse Pressure Is Associated with Rapid Cognitive Decline over 4 Years: A Population-Based Cohort Study

**DOI:** 10.3390/brainsci12121691

**Published:** 2022-12-09

**Authors:** Rong Zhou, Shan Wei, Yanyu Wang, Ling Gao, Liangjun Dang, Suhang Shang, Ningwei Hu, Wei Peng, Yi Zhao, Ye Yuan, Jingyi Wang, Jin Wang, Qiumin Qu

**Affiliations:** 1 Department of Neurology, The First Affiliated Hospital of Xi’an Jiaotong University, 277 West Yanta Rd, Xi’an 710061, China; 2 Huyi Hospital of Traditional Chinese Medicine, 304 Caotang Rd, Xi’an 710300, China; 3 Center for Brain Science, The First Affiliated Hospital of Xi’an Jiaotong University, 277 West Yanta Rd, Xi’an 710061, China

**Keywords:** cognitive decline, risk factor, pulse pressure, cohort study

## Abstract

Aiming to investigate the relationship between pulse pressure (PP) and cognitive decline, cognitively normal subjects from a community-based longitudinal cohort were followed-up for 4 years. The Mini-Mental State Examination (MMSE) was used to evaluate global cognitive function, and a ≥2-point decrease in the MMSE score from baseline was defined as cognitive decline. Restricted cubic spline, multivariable linear regression and logistic regression were used to investigate the relationship between PP and cognitive decline. A total of 1173 participants completed the follow-up, and 205 (17.5%) met the criteria for cognitive decline. Restricted cubic splines showed no nonlinear relationship between PP and ΔMMSE (P_overall_ = 0.037, P_non-linear_ = 0.289) or cognitive decline (P_overall_ = 0.003, P_non-linear_ = 0.845). Multivariable linear regression analysis showed that PP was positively related to ΔMMSE (b = 0.021, *p* = 0.020). Multivariable logistic regression analysis showed that PP was positively associated with cognitive decline (OR = 1.020, *p* = 0.023). A stratified analysis found an association between PP and cognitive decline in participants who were aged ≤65 years, male, and APOEε4 noncarriers and who had school education ≤6 years or hypertension. A sensitivity analysis after propensity-score matching did not alter our findings. These findings highlight that elevated PP is associated with rapid cognitive decline, particularly in males, middle-aged, low-educated, hypertensive individuals and APOEε4 noncarriers.

## 1. Introduction

According to the latest statistics from Alzheimer’s Disease International, approximately 55 million people worldwide suffered from dementia in 2021, and this number will increase to 78 million by 2030 [1]. Alzheimer’s disease (AD) is the most common cause of dementia and accounts for 50–70% of all dementias. There are 15.07 million people with AD in the population 60 years and older in China, and the prevalence of mild cognitive impairment (MCI) is 15.5%, i.e., 38.77 million people [2]. With no effective treatments for cognitive decline or dementia, identifying modifiable risk factors is a research priority [3].

Several studies have shown that hypertension plays an important role in many diseases, such as coronary heart disease and stroke [4]. However, the relationship between hypertension and cognitive impairment is still unclear, and it seems that the effect of hypertension may be complex, age-dependent and inconsistent [5]. Some studies revealed that middle-aged individuals with hypertension had higher risks of cognitive impairment later in life [6,7,8]. Other studies reached conflicting results about later-life hypertension: some suggested the deleterious impact of hypertension on cognition in older adults, while many others failed to find positive results [9,10,11,12].

The age-dependent effect of hypertension on cognition could be related to the impact of steady blood flow on cerebral autoregulation [13]. This regulation ability is also associated with pulse pressure (PP) [14], which is produced by the combined effect of stroke volume and the arterial circulatory properties that determine compliance and wave reflection [15]. Under normal physiological conditions, blood flows in an oscillating waveform to generate PP, which propagates along the circulatory system and helps regulate cerebral vascular endothelial function and cerebral perfusion [16]. Since PP has been implicated as an independent risk factor for cardiovascular disorders and stroke [17,18], it is of significance to detect the relationship between PP and cognitive decline.

Previous studies have found that widened pulse pressure was associated with neurodegenerative changes prior to the onset of dementia [19]. Another study found that elevated PP increased the risk of incident significant cognitive impairment [20]. In addition, it is suggested that high PP is associated with impairments of verbal learning, nonverbal memory, working memory and delayed recall [21]. The above studies suggest that high PP is related to cognitive impairment, but its relationship with cognitive decline is not yet clear. Some epidemiological studies of Black people and White people suggest that high PP is associated with significant cognitive decline [22], but other studies have reached different conclusions [23]. A study from China showed that higher PP is an independent factor in reducing the cognitive ability of middle-aged and elderly people but that it does not contribute to the rate of cognitive change [24]. These results suggest that the relationship between PP and cognitive decline should be studied further.

Based on the above findings, we hypothesized that elevated PP is associated with an increased risk of rapid cognitive decline. Therefore, we conducted a community-based longitudinal cohort study to investigate the relationship between PP and cognitive decline by following participants for 4 years.

## 2. Materials and Methods

### 2.1. Study Design

**Figure 1:**
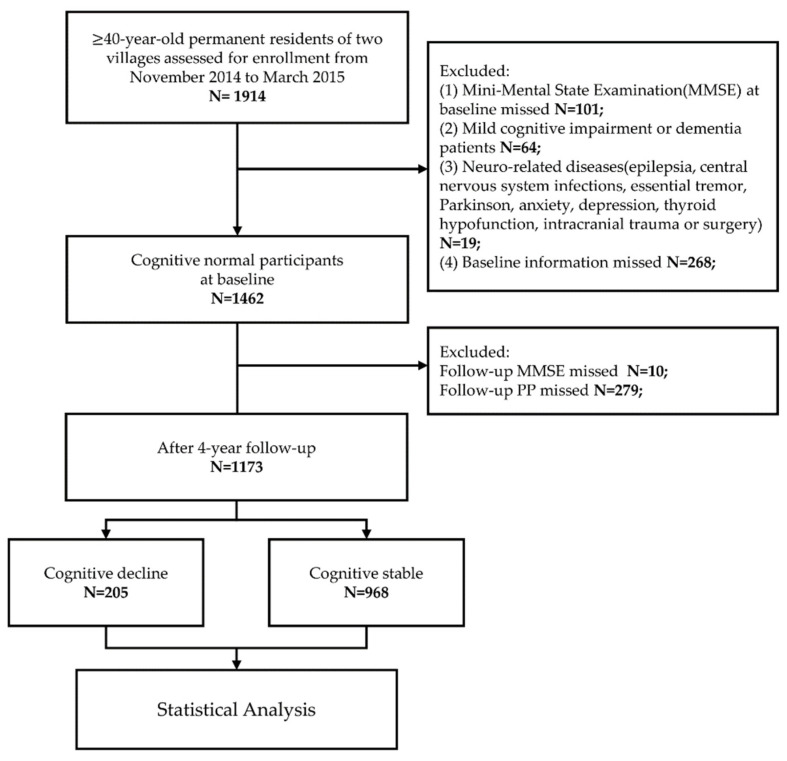
Flow chart of research design.

We designed a community-based cohort study. A total of 1462 participants with normal cognition were screened and followed up. Finally, 1173 participants were included, including 205 in the cognitive decline group and 968 in the cognitively stable group. Multivariable linear regression, multivariable logistic regression and restricted cubic spline (RCS) were performed to analyze the relationship between PP and cognitive decline (Figure 1).

### 2.2. Study Population

This was a population-based longitudinal cohort study. We used a stratified, multistage, cluster-sampling methodology to select Qubao and Bitou Village, Huyi District, Xi’an and Shaanxi Province, China, for recruiting the study population. The study was conducted from October 2014 through March 2015. The inclusion criteria for this study were as follows: (1) age over 40; (2) current residence in the village and having lived in the village for more than 3 years; and (3) willingness to participate in the study and sign informed consent. The exclusion criteria were as follows: (1) no Mini-Mental State Examination (MMSE); (2) evidence of MCI or dementia at the baseline investigation; (3) other neurological diseases that may affect cognitive function (including epilepsy, central nervous system infection, essential tremor, Parkinson’s disease, anxiety, depression, hypothyroidism, intracranial trauma or surgery); (4) severe cardiac, pulmonary, hematologic, hepatic or renal disease or tumors; and (5) other missing baseline information (Figure 1). This study was approved by the Ethical Review Committee of the First Affiliated Hospital of Xi’an Jiaotong University. All participants signed a written informed consent form.

### 2.3. Standard Interview and Follow-Up

All participants underwent a standardized face-to-face interview in the baseline investigation. The interviewers consisted of neurologists and medical students. All staff received at least one week of training by supervisors for the correct use of the unified questionnaires, standardized survey terms, assessment of cognition and community practice. The consistency between the examiners (kappa: 0.76–1) was evaluated afterward.

The standardized questionnaires asked for information about demographic data (age, sex and years of education), lifestyle (smoking, drinking and physical exercise habits), medical history (hypertension, diabetes, dyslipidemia, cardiovascular disease and cerebral vascular disease) and medication status. Additionally, all subjects received a physical examination (height, weight, pulse rate, blood pressure (BP), etc.), neurological examination and cognitive assessment.

All participants who completed the baseline survey were followed up in 2016 and 2018 using the same standardized face-to-face interview as in the baseline survey.

### 2.4. Cognitive Assessment

The MMSE was used to evaluate global cognition at both baseline and follow-ups in 2016 and 2018. The change in the MMSE score (△MMSE) was calculated as the baseline score subtracted from the follow-up score. Previous studies have suggested that a 2–4-point decrease in the MMSE score in an elderly population at long-term follow-up indicates a reliable cognitive change [25,26]. Considering the younger population and shorter follow-up period in this study, ΔMMSE ≥ 2 points was defined as cognitive decline, while a drop in the MMSE score of <2 points was defined as cognitively stable.

### 2.5. Blood Pressure Measurement

The BP of the right arm was measured twice while subjects were in the sitting position by uniformly trained nurses using a mercury sphygmomanometer (Shanghai Medical Instruments Co. Shanghai, China) that was calibrated regularly. Participants were asked to rest quietly for 10 min and to avoid strenuous exercise for 30 min prior to the measurement. The average of two measurements was recorded as the BP of each participant. PP was defined as the difference between systolic blood pressure (SBP) and diastolic blood pressure (DBP). Mean arterial pressure (MAP) was calculated as 1/3 SBP plus 2/3 DBP. For each participant, the mean value (PP_mean_) of the three PPs obtained from the baseline and follow-up surveys was included in the statistical analysis.

### 2.6. Laboratory Evaluation

Under the condition of fasting for more than 8 h, 10 mL of blood was drawn from each subject’s elbow vein, placed in a red-topped nonanticoagulation tube and sent to the biochemical laboratory of the First Affiliated Hospital of Xi’an Jiaotong University for biochemical evaluation. Fasting blood glucose (FBG), triglycerides (TG), total cholesterol (TC), low-density lipoprotein cholesterol (LDL-c) and high-density lipoprotein cholesterol (HDL-c) were measured enzymatically using a fully automated biochemical analyzer (C501, Roche, Sweden).

Five milliliters of fasting blood was placed in a purple-capped EDTA anticoagulant tube. The blood samples were centrifuged within 2 h at a centrifugal force of 1500 g for 10 min, and then the upper plasma and lower blood cells were separated and stored in a refrigerator at −80 °C for subsequent blood tests.

Genomic DNA was extracted from frozen EDTA-anticoagulated blood using a DNA extraction kit (Tiangen, Beijing, China) and used as a template to PCR amplify a 244 bp APOE DNA fragment containing two polymorphic sites at amino acid residues 112 and 158 [27]. All PCR products were tested by Sanger sequencing (Sangon, Shanghai, China) to confirm the APOE genotype as previously described. Participants were classified as APOEε4 carriers (APOE 2/4, APOE 3/4 and APOE 4/4) and APOEε4 noncarriers (APOE 2/2, APOE 2/3 and APOE 3/3).

### 2.7. Definition of Variates

The lack of physical exercise was defined as exercising less than 3 times a week for less than 30 min. Smoking was defined as being a current smoker. Diabetes was defined as a self-reported confirmed medical history, the use of medication for diabetes, FBG ≥ 7.0 mmol/L, random blood glucose concentration ≥ 11.1 mmol/L or glycosylated hemoglobin ≥ 6.5%. Dyslipidemia was defined as TC ≥ 5.18 mmol/L and/or TG ≥ 1.70 mmol/L and/or LDL-c ≥ 3.37 mmol/L. Hypertension was defined as a self-reported confirmed medical history, the use of antihypertensive drugs, SBP ≥ 140 mmHg and/or DBP ≥ 90 mmHg.

### 2.8. Statistical Analysis

Continuous normal and approximately normal variables are represented by $\bar{x}$ ± SD, continuous nonnormal variables are denoted by median (interquartile range) [M(IQR)], and categorical variables are expressed in proportions. Participants were divided into cognitively stable and cognitive decline groups based on whether the difference in their MMSE scores was greater than or equal to two, and the differences in population baseline characteristics between the two groups were compared. Student’s t-test or the Mann–Whitney U test was used for continuous variables, and Pearson’s chi-square test or Fisher’s exact test was used for categorical variables. A paired t-test was used to verify the differences between the 3 measurements of PP. 

RCS was performed using the R package rms [28] to explore whether there is a nonlinear relation between continuous PP and cognitive decline. The correlation between PP and ΔMMSE at 4 years was tested by the Pearson correlation test. Then, multivariable linear regression models were used to assess the relationship between continuous PP and ΔMMSE, and multivariable logistic regression models were used for continuous PP and cognitive decline. In the multivariable model, adjustments were made for age, sex, years of education, lack of physical activity, MAP or hypertension, diabetes, cardiovascular disease and APOEε4 carriage. In the subgroup analyses, we explored the role of each factor in the relationship between PP and cognitive decline by age, sex, education, APOEε4 carrier status and history of hypertension. 

For the sensitivity analysis, we examined the association between PP as a dichotomous variable and cognitive decline using data before and after propensity-score matching (PSM). One-to-one PSM was performed using a nearest-neighbor matching algorithm without replacement and a caliper of 0.2 standard deviations of the logit of the propensity score obtained from logistic regression with the R package MatchIt [29]. Standardized mean differences (SMDs) lower than 0.10 were considered acceptable.

In this study, for each regression model, residual analysis (Durbin–Watson test, histogram of residuals and QQ plot) was performed, and the residuals were independent and normal with equal variances. In addition, multicollinearity statistics (tolerance and variance inflation factors) were calculated to ensure a proper fit. All statistical analyses were performed using IBM SPSS Statistics version 25.0 (IBM, New York, NY, USA) and the R programming language version 4.1.3 [30], and *p* < 0.05 was considered to indicate significance.

## 3. Results

### 3.1. Characteristics of the Study Population at Baseline

At the baseline of the study, there were 1914 individuals enrolled. Of them, 101 did not complete BP measurements and the MMSE, 64 had MCI or dementia, 19 had neurological diseases that may affect cognitive function, and 268 lacked other baseline information. Finally, 1462 participants were enrolled at baseline.

The follow-ups were conducted in 2016 and 2018. There were 279 patients lost to follow-up, and 10 refused to finish the MMSE. Finally, a total of 1173 participants (80.2%) were included in the analysis (Figure 1).

During the 4-year follow-up, 205 (17.5%) met the criteria for cognitive decline. As shown in Table 1, the cognitive decline group was older (54.0 vs. 56.0, *p* = 0.003) and less educated (8.0 vs. 7.0, *p* = 0.001) than the cognitively stable group, as well as the overall study group. In addition, the MMSE score of the cognitively stable group was higher than that of the cognitive decline group (28 vs. 27, *p* < 0.001), while other characteristics were not significantly different between the two groups.

### 3.2. The Relationship between PP and Cognitive Decline

The paired *t*-test showed that there was no significant difference in PP between baseline and the first follow-up (difference 0.8 mmHg, 95% CI: −0.1–1.6, *p* = 0.059) or between baseline and the second follow-up (difference −0.6 mmHg, 95% CI −1.5–0.3, *p* = 0.169). To minimize random error and accurately reflect the true PP level of the population during the study period, the average value of the three PP measurements was included in the subsequent analysis. 

**Figure 2:**
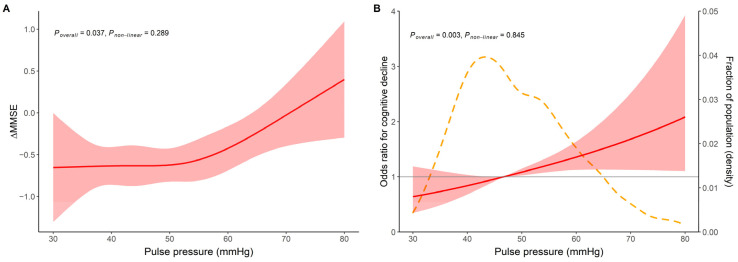
Restricted cubic spline for the relation between PP and ΔMMSE or cognitive decline. (**A**) PP and ΔMMSE; (**B**) PP and cognitive decline. Solid red line showed predicted value of ΔMMSE in Figure 2(B) and odds ratio in Figure 2(B) Shaded areas indicate 95% confidence intervals, and the fraction of the population with different levels of PP is indicated by the dashed yellow curve.

We first performed RCS to flexibly model and visualize the relation between continuous PP and the two dependent variables, either ΔMMSE (P_overall_ = 0.037, P_non-linear_ = 0.289) or cognitive decline (P_overall_ = 0.003, P_non-linear_ = 0.845), showing no nonlinear relationship (Figure 2). Pearson correlation analysis suggested that PP was positively related to ΔMMSE (r = 0.071, *p* = 0.014). 

To eliminate the influence of confounding factors, multivariable linear regression analysis was used to investigate the relationship between continuous PP and MMSE score changes. As shown in Table 2, PP was associated with ΔMMSE (b = 0.017, t = 2.451, *p* = 0.014). The relationship remained in Model 1 after adjusting for age and sex (b = 0.019, t = 2.315, *p* = 0.021). Additionally, PP was correlated with ΔMMSE in Model 2 after adjusting for education, lack of physical exercise, smoking, drinking, diabetes, cardiovascular disease, dyslipidemia, APOE ε4 carriage and MAP (b = 0.021, t = 2.339, *p* = 0.019), and in Model 3, where MAP was replaced with hypertension (b = 0.021, t = 2.323, *p* = 0.020).

Multivariable logistic regression analysis showed that PP was positively associated with cognitive decline (OR = 1.024, 95% CI = 1.010–1.037, *p* = 0.001) before adjustment. After adjustment for age and sex in Model 1, PP was positively associated with cognitive decline (OR = 1.016, 95% CI 1.000–1.032, *p* = 0.047). After further adjusting for education, lack of physical exercise, smoking, drinking, diabetes, cardiovascular disease, dyslipidemia, APOE ε4 carriage and MAP in Model 2 (OR = 1.019, 95% CI 1.001–1.037, *p* = 0.035) and replacing MAP with hypertension in Model 3, the effects remained significant (OR = 1.020, 95% CI 1.003–1.038, *p* = 0.023) (Table 3).

### 3.3. Subgroup Analysis of PP and Cognitive Decline

A stratified multivariable linear regression analysis was used to eliminate the influence of the covariates on the relationship between PP and ΔMMSE. As shown in Table 4, PP was positively associated with ΔMMSE in the age group ≤ 65 years (b = 0.021, t = 2.242, *p* = 0.025), in males (b = 0.036, t = 2.832, *p* = 0.005), in those with school education ≤ 6 years (b = 0.031, t = 2.088, *p* = 0.037), in APOEε4 noncarriers (b = 0.027, t = 2.796, *p* = 0.005), and in subjects with hypertension (b = 0.027, t = 2.346, *p* = 0.019). The same results on the relationship between PP and cognitive decline were found using multivariable logistic regression analysis in the age group ≤ 65 years (OR = 1.022, 95% CI: 1.002–1.042, *p* = 0.033), in males (OR = 1.031, 95% CI: 1.004–1.059, *p* = 0.025), in those with school education ≤ 6 years (OR = 1.026, 95% CI: 1.002–1.051, *p* = 0.031), in APOEε4 noncarriers (OR = 1.017, 95% CI: 1.001–1.034, *p* = 0.042), and in subjects with hypertension (OR = 1.030, 95% CI: 1.008–1.053, *p* = 0.007) (Table 5).

### 3.4. Sensitivity Analysis

**Figure 3:**
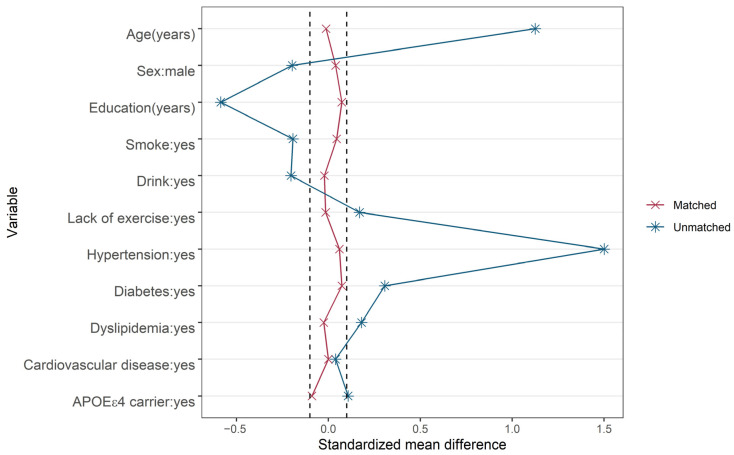
Love plot of standardized mean differences before and after propensity-score matching. The vertical dashed line indicates standardized mean difference = ±0.1.

In the sensitivity analysis, to explore the relation between abnormally high PP (usually considered to be above 60 mmHg) and cognitive decline, as well as to verify the robustness of the results, PP was converted to a dichotomous variable with a cutoff of 60 mmHg. PSM analysis was used to create an overall balance between the two groups. A total of 162 participants in the high-PP group were matched with the same number in the low-PP group as a control based on age, sex, education, smoking, drinking, lack of exercise, hypertension, diabetes, dyslipidemia, cardiovascular disease and APOE ε4 carriage. As shown in the Love plot, all SMDs were narrowed down to <10% after matching, which suggested that baseline characteristics between comparison groups obtained a better balance (Figure 3). We then conducted the same analysis as before using the data before and after PSM. The results were consistent with the main analysis (Table 6).

## 4. Discussion

In this prospective, population-based cohort study, we followed participants for 4 years. By doing so, we found that individuals with higher PP had a higher rate of cognitive decline and that PP was positively related to the drop in MMSE scores and cognitive decline. Stratified analysis showed that an association between PP and cognitive decline was found in subjects aged ≤65 years, in males, in those with school education ≤ 6 years, in APOEε4 noncarriers and in hypertensive subjects.

PP is the difference between SBP and DBP, and a higher PP indicates increased arterial stiffness and arteriosclerosis. PP is an independent risk factor for cardiovascular disease, but the relationship between PP and cognitive impairment has not been determined [31]. Some studies have observed a positive association between PP and cognitive impairment, and a high PP has been associated with impairments in global cognitive function and attention [32], memory function [33], episodic secondary memory performance and memory retrieval speed [23]. One study found a U-type association between PP and cognitive function [34], and another obtained a negative result between PP variability and cognitive function [35].

The present study is a large, population-based cohort study. We used a stratified, multistage, cluster-sampling methodology to select the study population, which made the population demographics consistent with the characteristics of Xi’an’s rural residents. To investigate the effects of PP on cognitive impairment, we excluded subjects with pre-existing cognitive impairment. By following changes in the MMSE score, we demonstrated that PP was associated with cognitive decline, even after eliminating confounding factors using several analysis methods.

For detecting preclinical cognitive changes, MMSE score declines are of more value than a diagnosis of MCI or dementia. The MMSE is a widely used neuropsychological screening instrument that evaluates global cognitive function in older adults. It can also be used to measure cognitive change over time [36], and a large number of studies have confirmed its good reliability and validity [25,26]. Preferably, the interpretation of individual changes should rely on norms for change with follow-up lengths and population characteristics similar to the ones that were actually used. A previous study, which included cognitively normal individuals aged 75 years and over, found that a change in the MMSE score of at least 2–4 points indicated a reliable change at the 90% confidence level over a mean period of 7.1 years [25]. Another study reported that a 2–3-point drop in the MMSE score reliably indicated changes among cognitively healthy older adults over a period of 4.5 years [26]. There were also findings that a reliable change was defined by differences of 3–4 points on the MMSE for long intervals [37,38]. Considering that the participants in our study were younger and underwent a shorter follow-up interval, which might suggest a slower rate of cognitive decline in our population, we used a drop in MSE score of ≥2 points to define cognitive decline.

The reason why PP was related to cognitive decline only in subjects aged ≤65 years, males, those with school education ≤ 6 years, those with hypertension and APOEε4 noncarriers was not determined. Since aging and female sex are risk factors for cognitive impairment [39,40], it has been found that female individuals or older individuals often have higher PP levels [41]. We suspected that, on the one hand, females and older adults exhibited less sensitivity to PP, and on the other hand, the effect of sex or age on cognition overlapped with the part caused by PP. Education is protective against cognitive decline by increasing cognitive reserve. Low education levels, typically associated with lower economic status and poorer health care, may lead to poor BP control and higher PP, which then accelerates cognitive decline [39]. As the impact of hypertension on cognition may rely on the effect of consistent steady blood flow on cerebral autoregulation [13], it is reasonable to hypothesize that people with high BP may be more sensitive to dramatic changes in BP over a short period of time, which would be caused by higher PP. In addition, a previous study found that favorable modifiable-risk profiles are related to a lower risk of dementia only in APOEε4 carriers [42]. As APOEε4 carriage is a strong risk factor for cognitive impairment [39], the influence of modifiable risk factors might be greater in the APOEε4-unrelated variant [43].

The mechanism by which higher PP contributes to cognitive decline has not been determined. We suspected that cognitive decline may result from cerebral perfusion and AD pathology. The brain has a relatively wide range of automatic BP regulations. The cerebral microcirculation is characterized by high flow and low impedance, and damage to the microcirculation from increased pulsatile energy due to increased PP is more pronounced in the brain [44]. In addition, the pathological remodeling of vessel walls caused by excessive PP elevation interferes with local blood flow steady-state and dynamic control and limits blood flow or hyperemic flow reserve, resulting in microvascular ischemia [45]. In addition, it was observed that high PP and mechanical stress led to a reduction in upstream endothelial dilatory reactivity, the barrier-dysfunction-associated loss of microvascular density and microhemorrhages in the mouse brain [46]. Together, these factors contribute to reduced cerebral perfusion, and it has been shown that hypoperfusion precedes the onset of cognitive impairment [47].

The main features of AD pathology are amyloid plaques composed of β-amyloid (Aβ) deposition and neurogenic fiber tangles composed of phosphorylated tau. A study reported that higher PP in middle age and old age was associated with tau-related markers and cognitive impairment [48]. In very elderly individuals, high PP was associated with elevated p-Tau and reduced Aβ_1-42_ in the cerebrospinal fluid, and dementia progressed more rapidly in those with higher PP [19]. This may be related to impaired blood–brain barrier dysfunction and reduced cerebral blood flow leading to impaired Aβ clearance [49]. Considering that plasma Aβ levels have been related to brain Aβ deposition, our previous study found that the elevation of PP is associated with increased amyloid-β_1-40_ and decreased soluble receptor of advanced glycation end-products among individuals not taking antihypertensive medication [50]. These results support the hypothesis that higher PP may accelerate Aβ pathology in AD.

This study still has limitations that need to be considered. First, only the MMSE was used to assess cognitive changes. Although the MMSE is a widely accepted cognitive measure suitable for large populations, the MMSE itself has the limitation that it is not sensitive enough to minor cognitive decline, and there is no precise cutoff value defining whether cognitive decline has occurred. Second, the follow-up of only 4 years was relatively short. Third, due to the smaller sample size, we did not analyze cognitive decline as MCI or dementia and did not diagnose cognitive decline due to AD or other diseases.

## 5. Conclusions

Elevated PP is associated with rapid cognitive decline, particularly in males, middle-aged, low-educated, hypertensive individuals and APOEε4 noncarriers.

## Figures and Tables

**Table 1:** Comparison of cognitive decline and cognitively stable groups.

	Total	Cognitive Decline	Cognitively Stable	*t, Z, or χ^2^*	*p*
	n = 1173	n = 205	n = 968		
Age, years M (IQR)	54.0 (15.0)	56.0 (15.0)	54.0 (14.0)	2.94	0.003
Male (n, %)	477 (40.7)	75.0 (36.6)	402 (41.5)	1.71	0.191
Education, years M (IQR)	8.0 (4.0)	7.0 (5.0)	8.0 (4.0)	3.46	0.001
Smoking (n, %)	331 (28.2)	56 (27.3)	275 (28.4)	0.10	0.752
Drinking (n, %)	163 (13.9)	27 (13.2)	136 (14.0)	0.11	0.741
Lack of exercise (n, %)	194 (16.5)	31 (15.1)	163 (16.8)	0.36	0.548
Dyslipidemia (n, %)	602 (51.3)	113 (55.1)	489 (50.5)	1.44	0.231
Diabetes (n, %)	142 (12.1)	30 (14.6)	112 (11.6)	1.49	0.222
Hypertension (n, %)	587 (50.0)	108 (52.7)	479 (48.5)	0.69	0.405
Cardiovascular disease (n, %)	68 (5.8)	14 (6.8)	54 (5.6)	0.49	0.486
Pulse rate, bpm M (IQR)	75.0 (10.0)	75.0 (10.0)	76.0 (10)	0.40	0.688
BMI, kg/m2 $\bar{x}$ ± SD	25.4 ± 3.1	25.5 ± 3.0	25.4 ± 3.2	0.39	0.697
APOEε4 carriers (n, %)	181 (15.4)	28 (13.7)	153 (15.8)	0.60	0.439
SBP, mmHg $\bar{x}$ ± SD	131.6 ± 17.7	133.5 ± 18.3	131.2 ± 17.6	1.65	0.099
DBP, mmHg $\bar{x}$ ± SD	82.2 ± 10.7	82.2 ± 10.6	82.2 ± 10.9	0.04	0.965
MAP, mmHg $\bar{x}$ ± SD	98.7 ± 12.1	99.3 ± 11.9	98.5 ± 12.2	0.83	0.407
TG, mmol/L M (IQR)	1.5 (1.0)	1.5 (1.0)	1.4 (1.0)	1.04	0.297
TC, mmol/L M (IQR)	5.0 (1.2)	5.1 (1.3)	4.9 (1.2)	1.05	0.315
HDL, mmol/L M (IQR)	1.35 (0.4)	1.4 ± (0.4)	1.4 (0.4)	0.30	0.764
LDL, mmol/L M (IQR)	3.2 (1.1)	3.3 (1.2)	3.2 (1.1)	0.92	0.357
FBG, mmol/L M (IQR)	5.4 (0.8)	5.4 (0.8)	5.4 (0.8)	0.65	0.516
MMSE, M (IQR)	27 (4)	28 (4)	27 (3)	4.136	<0.001

Abbreviations: M (IQR), median (interquartile range); BMI, body mass index; APOE, apolipoprotein E; SBP, systolic blood pressure; DBP, diastolic blood pressure; MAP, mean arterial pressure; PP, pulse pressure; TG, triglycerides; TC, total cholesterol; HDL, high-density lipoprotein; LDL, low-density lipoprotein; FBG, fasting blood glucose; MMSE, The Mini-Mental State Examination.

**Table 2:** Multivariable linear regression analysis of PP and ΔMMSE.

	B	95% CI for B		Std. Error	t	*p*
		Lower	Upper			
Unadjusted	0.017	0.003	0.031	0.007	2.451	0.014
Model 1	0.019	0.003	0.034	0.008	2.315	0.021
Model 2	0.021	0.003	0.038	0.009	2.339	0.019
Model 3	0.021	0.003	0.038	0.009	2.323	0.020

PP was included in this analysis as a continuous variable. Model 1, adjusted for age and sex. Model 2, adjusted for age, sex, education, lack of physical exercise, smoking, drinking, diabetes, cardiovascular disease, dyslipidemia, APOE ε4 carriage and MAP. Model 3, adjusted for age, sex, education, lack of physical exercise, smoking, drinking, diabetes, cardiovascular disease, dyslipidemia, APOE ε4 carriage and hypertension. Abbreviations: CI, confidence interval; ΔMMSE, The Mini-Mental State Examination score changes.

**Table 3:** Multivariable logistic regression analysis of PP and cognitive decline.

	B	S.E	Wald	*p*	OR	95% CI for OR	
						Lower	Upper
Unadjusted	0.023	0.007	11.746	0.001	1.024	1.010	1.037
Model 1	0.016	0.008	3.943	0.047	1.016	1.000	1.032
Model 2	0.019	0.009	4.439	0.035	1.019	1.001	1.037
Model 3	0.020	0.009	5.176	0.023	1.020	1.003	1.038

PP was included in this analysis as a continuous variable. Model 1, adjusted for age and sex. Model 2, adjusted for age, sex, education, lack of physical exercise, smoking, drinking, diabetes, cardiovascular disease, dyslipidemia, APOE ε4 carriage and MAP. Model 3, adjusted for age, sex, education, lack of physical exercise, smoking, drinking, diabetes, cardiovascular disease, dyslipidemia, APOE ε4 carriage and hypertension. Abbreviations: OR, odds ratio.

**Table 4:** Subgroup analysis of relationship between PP and ΔMMSE.

Subgroups	B	95%CI for B		t	*p*
		Lower	Upper		
Age ^a^					
≤65 years	0.021	0.003	0.040	2.242	0.025
>65 years	0.010	−0.031	0.051	0.470	0.639
Sex ^b^					
Male	0.036	0.011	0.061	2.832	0.005
Female	0.011	−0.014	0.035	0.866	0.387
School education ^c^					
≤6 years	0.031	0.002	0.060	2.088	0.037
>6 years	0.013	−0.008	0.035	1.255	0.210
APOE ε4 carrier ^d^					
Yes	−0.017	−0.067	0.033	0.669	0.505
No	0.027	0.008	0.046	2.796	0.005
Hypertension ^e^					
Yes	0.027	0.004	0.050	2.346	0.019
No	0.006	−0.025	0.037	0.385	0.700

PP was included in this analysis as a continuous variable. ^a^ Fully adjusted for sex, education, smoking, drinking, lack of physical exercise, BMI, dyslipidemia, diabetes, cardiovascular disease, APOEε4 carriage and MAP. ^b^ Fully adjusted for age, education, smoking, drinking, lack of physical exercise, BMI, dyslipidemia, diabetes, cardiovascular disease, APOEε4 carriage and MAP. ^c^ Fully adjusted for age, sex, smoking, drinking, lack of physical exercise, BMI, diabetes, cardiovascular disease, dyslipidemia, APOEε4 carriage and MAP. ^d^ Fully adjusted for age, sex, education, lack of physical exercise, smoking, drinking, BMI, diabetes, cardiovascular disease, dyslipidemia and MAP. ^e^ Fully adjusted for age, sex, education, lack of physical exercise, smoking, drinking, APOE ε4 carriage and MAP.

**Table 5:** Subgroup analysis of relationship between PP and cognitive decline.

Subgroups	No. of Events (%)	OR	95%CI		*p*
			Lower	Upper	
Age ^a^					
≤65 years	159 (16.2)	1.022	1.002	1.042	0.033
>65 years	46 (24.0)	1.019	0.987	1.051	0.243
Sex ^b^					
Male	75 (15.7)	1.031	1.004	1.059	0.025
Female	130 (18.7)	1.011	0.988	1.035	0.351
School education ^c^					
≤6 years	102 (21.2)	1.026	1.002	1.051	0.031
>6 years	103 (14.9)	1.006	0.978	1.035	0.668
APOE ε4 carrier ^d^					
Yes	28 (15.5)	0.995	0.949	1.042	0.818
No	177 (17.8)	1.017	1.001	1.034	0.042
Hypertension ^e^					
Yes	108 (18.4)	1.030	1.008	1.053	0.007
No	97 (16.6)	0.993	0.960	1.027	0.692

PP was included in this analysis as a continuous variable. ^a^ Fully adjusted for sex, education, dyslipidemia, APOE ε4 carriage and MAP. ^b^ Fully adjusted for age, education, smoking, drinking, dyslipidemia, APOE ε4 carriage and MAP. ^c^ Fully adjusted for age, sex, lack of physical exercise, smoking, drinking, BMI, diabetes, dyslipidemia, APOE ε4 carriage and MAP. ^d^ Fully adjusted for age, sex and education. ^e^ Fully adjusted for age, sex, education, lack of physical exercise, smoking, drinking, APOE ε4 carriage and MAP.

**Table 6:** Sensitivity analysis of relationship between PP and cognitive decline.

	Linear Regression Analysis				Logistic Regression Analysis			
	OR	95%CI		*p*	B	95%CI		*p*
Total population								
Unadjusted	1.932	1.310	2.812	<0.001	0.525	0.106	0.943	0.014
Model 1	1.594	1.048	2.394	0.027	0.524	0.076	0.971	0.022
Model 2	1.631	1.049	2.513	0.028	0.530	0.061	0.999	0.027
Model 3	1.622	1.044	2.498	0.029	0.491	0.024	0.957	0.039
After PSM								
Unadjusted	1.807	1.058	3.130	0.032	0.611	0.003	1.219	0.049
Model 4	1.808	1.059	3.133	0.032	0.613	0.005	1.221	0.048

PP was included in this analysis as a dichotomous variable. Model 1, adjusted for age and sex. Model 2, adjusted for age, sex, education, lack of physical exercise, smoking, drinking, diabetes, cardiovascular disease, dyslipidemia, APOE ε4 carriage and MAP. Model 3, adjusted for age, sex, education, lack of physical exercise, smoking, drinking, diabetes, cardiovascular disease, dyslipidemia, APOE ε4 carriage and hypertension. Model 4, adjusted for distance.

## Data Availability

The data presented in this study are available on request from the corresponding author. The data are not publicly available due to privacy restrictions.
